# Exploring the Role of Media Sources on COVID-19–Related Discrimination Experiences and Concerns Among Asian People in the United States: Cross-Sectional Survey Study

**DOI:** 10.2196/21684

**Published:** 2020-11-06

**Authors:** Nan Yu, Shuya Pan, Chia-chen Yang, Jiun-Yi Tsai

**Affiliations:** 1 Nicholson School of Communication and Media University of Central Florida Orlando, FL United States; 2 School of Journalism and Communication Renmin University of China Beijing China; 3 Center of Journalism and Social Development Renmin University of China Beijing China; 4 School of Educational Foundations, Leadership and Aviation Oklahoma State University Stillwater, OK United States; 5 School of Communication Northern Arizona University Flagstaff, AZ United States

**Keywords:** COVID-19, discrimination, Asians, Asian Americans, media source, social media, prejudice

## Abstract

**Background:**

Media coverage and scholarly research have reported that Asian people who reside in the United States have been the targets of racially motivated incidents during the COVID-19 pandemic.

**Objective:**

This study aimed to examine the types of discrimination and worries experienced by Asians and Asian Americans living in the United States during the pandemic, as well as factors that were associated with everyday discrimination experience and concerns about future discrimination that the Asian community may face.

**Methods:**

A cross-sectional online survey was conducted. A total of 235 people who identified themselves as Asian or Asian American and resided in the United States completed the questionnaire.

**Results:**

Our study suggested that up to a third of Asians surveyed had experienced some type of discrimination. Pooling the responses “very often,” “often,” and “sometimes,” the percentages for each experienced discrimination type ranged between 14%-34%. In total, 49%-58% of respondents expressed concerns about discrimination in the future. The most frequently experienced discrimination types, as indicated by responses “very often” and “often,” were “people act as if they think you are dangerous” (25/235, 11%) and “being treated with less courtesy or respect” (24/235, 10%). About 14% (32/235) of individuals reported very often, often, or sometimes being threatened or harassed. In addition, social media use was significantly associated with a higher likelihood of experiencing discrimination (β=.18, *P*=.01) and having concerns about future episodes of discrimination the community may face (β=.20, *P*=.005). Use of print media was also positively associated with experiencing discrimination (β=.31, *P*<.001).

**Conclusions:**

Our study provided important empirical evidence regarding the various types of discrimination Asians residing in the United States experienced or worried about during the COVID-19 pandemic. The relationship between media sources and the perception of racial biases in this group was also identified. We noted the role of social media in reinforcing the perception of discrimination experience and concerns about future discrimination among Asians during this outbreak. Our results indicate several practical implications for public health agencies. To reduce discrimination against Asians during the pandemic, official sources and public health professionals should be cognizant of the possible impacts of stigmatizing cues in media reports on activating racial biases. Furthermore, Asians or Asian Americans could also be informed that using social media to obtain COVID-19 information is associated with an increase in concerns about future discrimination, and thus they may consider approaching this media source with caution.

## Introduction

### Background

As of September 2020, COVID-19 has infected approximately 6.94 million people in the United States and caused more than 200,000 deaths in the country [1]. Globally, over 31 million people had been infected with COVID-19, making it one of the worst pandemics in human history [1,2]. It is common for people to seek the origin of a pandemic and focus on responsibility and assign blame during an outbreak [3]. Media reports and nonprofit organizations have alerted the Asian community in the United States regarding the rise of anti-Asian hate crimes since the start of this pandemic [4-6]. In late March, the Federal Bureau of Investigation also warned this community about a surge in hate crimes during the pandemic and alerted law enforcement agencies to pay closer attention to bias-motivated crimes [7,8].

Several empirical studies have indicated that Asians residing in the United States are vulnerable to racist behaviors during the outbreak of an infectious disease like COVID-19. Dhanani and Franz [9] conducted a national survey of US adults in March 2020 to study COVID-19–related discriminatory behaviors, including avoiding ordering from restaurants with primarily Asian employees, limiting interactions with Asian customers or coworkers, or intentionally moving away from an Asian individual while in a public place. Their study found that 42% of the 1141 people surveyed engaged in at least one discriminatory behavior toward people of Asian descent [9]. Another study showed that racism against Asian groups, which is associated with the outbreak of an infectious disease, is not a new phenomenon but has happened several times throughout American history [7]. Meanwhile, Asians across the globe were also found to experience xenophobia and stigmatization during the SARS (severe acute respiratory syndrome) outbreak in 2003 [10].

At the same time, according to several news reports, Asians and people of Asian descent who reside in the United States had been targets of blame, partly because the virus was first reported in Wuhan, China [11,12]. In addition, US President Donald Trump and other political officials have publicly referred to COVID-19 as “Wuhan virus,” “Chinese virus,” and “Kung Flu,” causing a surge in racist behavior against Asians [5,10,13]. The Asian Pacific Policy and Planning Council received approximately 1500 reports on anti-Asian hate incidents from 45 states during a 4-week period from March to April 2020 [6]. Although there is no existing research directly comparing the prevalence of anti-Asian incidents before and after the COVID-19 outbreak, both academic research and news reports have shown that the Asian community experienced a high level of verbal harassment, shunning, physical assaults, workplace discrimination, being barred from transportation, and being turned away by businesses [5-7,12,13]. The Center of Public Integrity [5] suggested that the national mental health support organization Crisis Text Line [14] received significantly more texts from people of Asian descent after President Trump called the coronavirus “Chinese virus” in late March 2020. During the same period, Reny and Barreto [15] found that Google searches for “Chinese virus” and “Kung Flu” increased dramatically.

Given the current social environment, it is meaningful to study the discrimination experienced by people of Asian descent living in the United States during the COVID-19 pandemic. Recently, scholars have studied relevant topics in the COVID-19 context, such as the impact of discrimination against Asians on mental health [10]; social media usage and anti-Asian sentiments among Caucasians [13]; and anti-Asian attitudes and behaviors in the United States [9].

The rise of social media has enabled it to powerfully influence people’s racism-related beliefs and actions. In a recent study, Davidson and Farquhar [16] showed that social media can serve as a major news source and contribute to prejudicial attitudes toward immigrants, refugees, and transgender people. Specifically relating to COVID-19, Croucher et al [13] found that users who perceived information on social media to be accurate and fair were more likely to believe that Chinese Americans could pose realistic threats (eg, threats to physical and material well-being) and symbolic threats (eg, threats to morals, values, and beliefs). Additionally, Ziems et al [17], in a preprint publication, revealed that the presence of anti-Asian hate speech on Twitter was more prevalent than counter-hate messages during the COVID-19 crisis. For Asians living in the United States, being exposed to large amounts of unscreened racist messages on social media could greatly affect their perceptions of discrimination. In fact, a recent study has shown that engagement with social media during the pandemic was associated with worry about discrimination among the Asian individuals surveyed [18].

According to cultivation theory [19], traditional media use could effectively construct a social reality as portrayed by the media sources among viewers. Being exposed to increasing racism-related media content during the COVID-19 pandemic, Asians may form the belief that anti-Asian racism is a salient concern in the United States, leading them to become more sensitive to the discrimination they experience in their daily lives as well as become more worried about the discrimination they may encounter in the future.

Scholars have noted that media exposure can contribute to beliefs and behaviors associated with social biases [16,20,21]. Research also showed that the media can reinforce the marginalization of ethnic minorities and stigmatized groups by portraying them as primary health threats to others [22]. However, previous research mainly documented how racism-related mainstream media coverage (eg, newspapers and TV) could cultivate dominant groups’ prejudicial attitudes toward racial minorities. Little is known about how exposure to different media sources could relate to the prejudiced/discriminated groups’ experiences and perceptions, which arguably reflect beliefs associated with social biases from the opposite perspective (ie, the perspective from the prejudiced and discriminated).

To advance these lines of research and create tailored interventions to promote the well-being of Asian people, our study aimed to examine the types of discrimination Asians in the United States have experienced and worried about during the pandemic, as well as how exposure to various traditional media and social media sources could influence experienced and expected future discrimination in this group during the COVID-19 pandemic. Our study is one of the first attempts to focus on Asians residing in the United States during the pandemic and examine factors influencing their experiences of discrimination.

### Aims of This Study

Racism refers to a type of ideology in which certain racial groups are viewed as superior to others [23], and racial biases are negative attitudes and beliefs of outgroup members [23-25]. Discrimination is described as unfair treatment and harassment caused by racial biases [24]. In this study, we focused on everyday discrimination, which refers to discriminative events that happen repeatedly in a variety of contexts, such as being treated disrespectfully, being shunned, being offered poor service, or being verbally or physically harassed [26]. The Everyday Discrimination Scale [24] adopted in this study is among the most widely used instruments to evaluate this type of discrimination [26].

We distinguished between two dimensions of discrimination: (1) experiences of discrimination and (2) concerns about future discrimination. Experiences of discrimination refer to people’s perceptions of the various forms of anti-Asian incidents encountered during COVID-19, whereas concerns related to future discrimination refers to the extent to which people worried about discrimination that might happen in the future. Our first research question focused on the prevalence of experiences related to discrimination and concerns about future discrimination among Asians who resided in the United States during the pandemic. Our second research question focused on examining the relationship between media sources and Asian participants’ experience of discrimination and concerns about future discrimination.

## Methods

### Sampling

Upon institutional review board (IRB) approval, a cross-sectional online survey was generated on Qualtrics and then distributed to eligible participants via Amazon Mechanical Turk (MTurk). MTurk subject pools have revealed proven advantages in recruiting participants during ongoing social events [27-29]. We posted our Qualtrics survey link on the MTurk platform, through which participants can join the study. MTurk helped us reach eligible participants, and responses from participants were stored in Qualtrics.

Eligible participants included individuals aged ≥18 years who identified as Asian and lived in the United States when the data were collected. To ensure that participants paid sufficient attention during survey completion and to exclude bots and responses from server farms, we included an attention check question in the survey. Specifically, the question, which was placed randomly within the questionnaire, asked participants to select a designated answer (eg, please select “never” here to show that you are paying attention); those who failed to select the designated answer were excluded from the study.

Data collection was completed in the first 2 weeks of May 2020. At that time, there was a significant number of confirmed COVID-19 cases in the United States, but some states had loosened up the stay-at-home orders. A total of 235 people who identified themselves as Asians or Asian Americans and resided in the United States completed the questionnaire. The sample size for this study was determined by an a priori estimate utilizing G*Power, version 3.1 (Heinrich-Heine-Universität Düsseldorf) [30]. With the regression effect size (R^2^) of 0.15, 95% power, and 14 predictors, the minimum sample size was calculated to be 153. In total, 202 participants were included in the regression analyses.

### Independent Variable: Media Source

The respondents were asked to indicate how often they obtained news regarding COVID-19 from different media sources. The items were rated on a 4-point scale (1=never to 4=often). Responses indicated media usage as follows: (1) newspapers or magazines (mean 1.69, SD 0.92), (2) radio (mean 1.85, SD 0.97), (3) TV (mean 2.50, SD 1.01), (4) social media (mean 3.22, SD 0.92), and (5) news websites or apps (mean 3.31, SD 0.88).

### Dependent Measures

#### Experience of Discrimination

Experience of discrimination was assessed by asking participants how often they encountered different forms of unfair treatment since the COVID-19 outbreak. The measurement items were adapted from the Everyday Discrimination Scale [24], which is a widely used instrument to evaluate discrimination [26].The items were rated on a 5-point scale (1=never to 5=very often). This item was worded as follows: “Since the COVID-19 outbreak, in your day-to-day life, how often have any of the following things happened to you: (1) you are treated with less courtesy or respect than other people; (2) people act as if they think you are dangerous; (3) people act as if they are afraid of you; (4) you receive poorer service than other people; (5) you are threatened or harassed.” Responses were averaged to indicate the overall experiences of discrimination participants perceived (Cronbach α=.94, mean 1.93, SD 0.98).

#### Concerns About Future Discrimination

This item measures the degree to which individuals worry about discrimination they may experience in the future. The same set of items in the Everyday Discrimination Scale [24] were used by changing them to future tenses (eg, “how often do you worry that you will be treated with less courtesy or respect than other people?”). Items were averaged to show the overall level of concerns about future discrimination (Cronbach α=.96, mean 2.61, SD 1.18).

### Data Analysis

Data were analyzed with SPSS Statistics 25 (IBM Corp). Descriptive statistics were used to investigate the prevalence of different types of discriminatory experiences Asians have experienced or expected to encounter in the future. Two hierarchical regression analyses were used to investigate factors that were associated with experienced everyday discrimination and concerns about future discrimination, respectively. Model 1 included demographic variables such as age, years living in the United States, sex, being Chinese, being a visitor (ie, not a US citizen or permanent resident), education level, employment status, income, and political orientation. Model 2 included the variables in Model 1 as well as different media sources. Some demographic variables were recoded for the regression analyses (sex: male=1, female=0; being Chinese: yes=1, no=0; visitor: visitor=1; US citizen and permanent resident=0; and employment status: employed=1; no=0). Age and years living in the United States were measured as continuous variables. Education level ranged from 1=<high school degree to 7=doctoral degree; income ranged from 1=<$10,000 to 12=≥$150,000; and political orientation varied from 1=very liberal to 7=very conservative. Assumptions, including normality, homoscedasticity, and multicollinearity, were checked before regression tests were performed. All assumptions were met. For the multicollinearity check, variance inflation factor values ranged between 1-2 and far below 10.

## Results

### Participant Demographics

In total, 235 individuals completed the survey. The participants’ age ranged from 18 to 73 years (mean 32.87, SD 10.95 years). Participants from 35 out of 50 states in the United States comprised the sample and had lived in the country for an average of 23.56 years (SD 12.95). Approximately 91% (n=212) of the participants reported being a US citizen or a permanent resident. About 35% (n=79) identified themselves as Chinese; the remainder were from other Asian countries.

As seen in Table 1, approximately half of the participants (n=122, 52%) identified themselves as male; 48% (n=112) were female. Further, 55% (n=128) of the participants reported being single, 42% (n=98) reported being married or being in a domestic partnership, and 3% (n=7) reported being widowed or divorced. About 6% (n=14) had an education level below college; 68% (n=160) reported having some college education, an associate degree, or a bachelor’s degree; and 26% (n=60) had earned a master’s or doctoral degree.

In total, 32% (n=72) of the participants were unemployed, 1% (n=3) was retired, and 67% (n=152) were employed on a part- or full-time basis at the time of data collection. Further, 23% (n=51) reported their household income as being <$39,999 in 2019, 49% (n=92) between $40,000 to $99,999, and 29% (n=64) >$100,000. In addition, 57% (n=126) of the participants reported that their family income had been impacted by the COVID-19 outbreak.

We also collected data on the participants’ political beliefs and orientations: 18% (n=48) were Republicans, 47% (n=110) were Democrats, and 25% (n=58) were Independent. More than half of the participants (n=113, 52%) reported being liberal or very liberal, 27% (n=59) classified themselves as moderate, and 21% (n=46) were conservative or very conservative (Table 1).

**Table 1:** Participants’ demographics (N=235).

Variable		Participants
**Age (years)**		
	Mean (SD)	32.87 (10.95)
	Range	18-73
**Sex, n (%)**		
	Male	122 (52.1)
	Female	112 (47.9)
**Years in the United States**		
	Mean (SD)	23.56 (12.95)
	Range	1-66
**Education^a^, n (%)**		
	Less than college	14 (5.6)
	Some college or college degree	160 (68.4)
	Graduate degree	60 (25.6)
**Chinese, n (%)**		
	Yes	79 (34.8)
	No	148 (65.2)
**Citizen or permanent resident, n (%)**		
	Yes	212 (91)
	No	21 (9)
**Annual household income^a^, n (%)**		
	≤$39,999	51 (22.8)
	$40,000-$99,999	92 (48.7)
	≥$100,000	64 (28.6)
**Income impacted by COVID-19, n (%)**		
	Yes	126 (57.3)
	No	94 (42.7)
**Marital status, n (%)**		
	Single	128 (54.9)
	Married or have a domestic partner	98 (42.1)
	Widowed	2 (0.9)
	Divorced	5 (2.1)
**Employment, n (%)**		
	Part-time or full-time	152 (67)
	Not employed	72 (31.7)
	Retired	3 (1.3)
**Party affiliation, n (%)**		
	Republican	48 (18.3)
	Democrat	110 (46.8)
	Independent	58 (24.7)
**Political orientation^a^, n (%)**		
	Liberal	113 (51.8)
	Moderate	59 (27.1)
	Conservative	46 (21.1)

^a^Regrouped for this table.

### Prevalence of Experienced Discrimination

Our first question asked about the prevalence of various types of discrimination experienced by Asians in the United States (Table 2). Pooling the responses “very often” and “often,” each experienced discrimination type ranged from 5% to 11%. The most frequently experienced discrimination types included “people act as if they think you are dangerous” (n=25, 11%); “being treated with less courtesy or respect” (n=24; 10%); followed by “received poorer service than other people” (n=20, 9%), and “people act if they are afraid of you” (n=17, 7%). Compared to the other four types of discriminatory experiences, encounters where individuals were threatened or harassed took place less frequently; only 5% (n=11) of participants experienced it “very often” or “often,” 9% (n=21) reported “sometimes,” and 86% (n=203) answered “rarely” or “never.” When the responses “very often,” “often,” and “sometimes” were pooled, each discrimination type was experienced by 14%-34% of the sample.

**Table 2:** Experience of discrimination among survey participants (N=235).

Item	Very often, n (%)	Often, n (%)	Sometimes, n (%)	Rarely, n (%)	Never, n (%)
You are treated with less courtesy or respect than other people.	11 (4.7)	13 (5.5)	48 (20.4)	67 (28.5)	96 (40.9)
People act as if they think you are dangerous.	7 (3.0)	18 (7.7)	55 (23.4)	50 (21.3)	105 (44.7)
People act as if they are afraid of you.	6 (2.6)	11 (4.7)	60 (25.5)	52 (22.1)	106 (45.1)
You receive poorer service than other people	4 (1.7)	16 (6.8)	43 (18.3)	59 (25.1)	113 (48.1)
You are threatened or harassed.	3 (1.3)	8 (3.4)	21 (8.9)	51 (21.7)	152 (64.7)

### Prevalence of Concerns About Future Discrimination

In terms of concerns about future experiences of discrimination, a higher percentage of the participants answered “very often,” “often,” or “sometimes” for the five discrimination types listed in Table 3. The results also showed that participants were generally concerned about the given forms of discrimination at similar levels. The pooled percentages of “very often” and “often” for each discrimination type ranged from 25% to 27%. The discrimination types of most frequent concern were “will be treated with less courtesy or respect than other people” (n=64, 27%) and “will receive poorer service than other people” (n=62, 26%). When the response “sometimes” was included, the range increased to 49%-58% (Table 3). 

**Table 3:** Concerns about future discrimination among survey participants (N=235).

Item	Very often, n (%)	Often, n (%)	Sometimes, n (%)	Rarely, n (%)	Never, n (%)
You will be treated with less courtesy or respect than other people.	18 (7.7)	46 (19.6)	71 (30.2)	46 (19.6)	54 (23.0)
People will act as if they think you are dangerous.	20 (8.5)	39 (16.6)	67 (28.5)	45 (19.1)	64 (27.2)
People will act as if they are afraid of you	17 (7.2)	43 (18.3)	63 (26.8)	48 (20.4)	64 (27.2)
You will receive poorer service than other people	17 (7.2)	45 (19.1)	62 (26.4)	47 (20.0)	64 (27.2)
You will be threatened or harassed.	19 (8.1)	40 (17.0)	57 (24.3)	52 (22.1)	67 (28.5)

### Factors Associated With Discrimination Experience and Concerns

Another question examined factors that may be associated with the experience of discrimination or concerns about future discrimination. The two sets of hierarchical regression analyses resulted in two significant models for experience of discrimination [R^2^=.183, *F*_14,187_=3.00, *P*<.001] and concerns about future discrimination [R^2^=.166, *F*_14,187_=2.66, *P*=.001]. Tables 4 and 5 demonstrate the results associated with these tests.

Our analyses revealed that age was negatively associated with both experience of discrimination (β=–.18, *P*=.03) and concerns about future discrimination (β=–.26, *P*=.003). This showed that younger people were more likely to experience discrimination and worry about future discrimination they may face. The length of residing in the United States was positively associated with both experience of discrimination (β=.20, *P*=.02) and concerns about future discrimination (β=.21, *P*=.02). This shows that the longer people lived in the United States, the more likely they felt discriminated or worried about how they might be treated in the future. Among different Asian ethnic groups residing in the United States, Chinese people were more likely to experience discrimination (β=.18, *P*=.01) or have concerns about future discrimination than other Asian groups (β=.15, *P*=.04).

Individuals who often used print (β=.31, *P*<.001) or social media (β=.18, *P*=.01) to obtain COVID-19–related news reported having experienced more discrimination. Using social media for COVID-19 was also positively associated with greater concerns about future discrimination (β=.20, *P*=.005).

**Table 4:** Hierarchical regression analysis for variables relating to experience of discrimination. Italics indicates significant results.

Step and variable		Model 1				Model 2				R^2^ change		*F* change		*P* value	
		β^a^		*P* value		β^a^		*P* value							
**Step 1: Demographic variables**										.077		1.77		.08	
	Age		–.13		.14		–*.18*		*.03*						
	Years in the United States		*.18*		*.045*		*.20*		*.02*						
	Sex		–.00		.96		–.02		.81						
	Chinese		*.19*		*.01*		*.18*		*.01*						
	Visitor		–.01		.91		–.04		.54						
	Education		.04		.59		.06		.42						
	Employment		–.04		.59		–.03		.64						
	Income		–.08		.31		–.05		.47						
	Political orientation		.05		.52		.09		.26						
**Step 2: COVID-19 media source**										.107		4.88^b^		.001	
	Print		—^c^		—		*.31*		*<.001*						
	Radio		—		—		.02		.79						
	TV		—		—		–.07		.37						
	News website or app		—		—		–.05		.48						
	Social media		—		—		*.18*		*.013*						

^a^All beta coefficients are standardized regression coefficients.

^b^F change is significant at *P*<.001.

^c^Not applicable.

**Table 5:** Hierarchical regression analysis for variables relating to concerns about future discrimination. Italics indicates significant results.

Step and variable			Model 1					Model 2					*R*^2^ change			*F* change			*P* value
			β^a^		*P* value		β			*P* value									
**Step 1: Demographic variables**												.114			2.74			.005	
	Age	–*.27*		*.002*		–*.26*			*.003*										
	Years in the United States	*.17*		*.047*		*.21*			*.02*										
	Sex	–.03		.73		–.04			.56										
	Chinese	*.15*		*.04*		*.15*			*.04*										
	Visitor	–.03		.68		–.04			.55										
	Education	–.01		.89		–.02			.83										
	Employment	–.08		.29		–.07			.37										
	Income	.03		.71		.03			.71										
	Political orientation	–.11		.11		–.10			.17										
**Step 2: COVID-19 media source**												.052			2.34			.04	
	Print	—^b^		—		.07			.39										
	Radio	—		—		-.01			.92										
	TV	—		—		.04			.59										
	News website or app	—		—		.06			.38										
	Social media	—		—		*.20*			*.005*										

^a^All beta coefficients are standardized regression coefficients.

^b^Not applicable.

## Discussion

### Principal Findings

This study examined what types of discrimination Asians residing in the United States have experienced and worried about during the pandemic, as well as factors that were associated with everyday discrimination experience and concerns about future discrimination that the Asian community may face. Our study suggested that a substantial part of Asians surveyed had experienced and worried about some type of discrimination. The relationship between media sources and the perception of racial biases among this group was also identified. Our study discovered the significant role of social media use in the higher likelihood of experiencing discrimination and having concerns about future discrimination. Use of print media was also positively associated with experiencing discrimination. The detailed discussion of these findings is presented below.

First, our study provided additional empirical evidence suggesting that a substantial part of the Asian community in the United States had experienced and worried about some type of discrimination at least part of the time (as indicated by the response “sometimes”). These findings complemented what the Center for Public Integrity Poll had suggested: 1 out of 4 people in the United States said they would be concerned about close contact with someone of Asian ancestry in public during the pandemic [5,12]. Additionally, about 14% (32/235) of the participants reported very often, often, or sometimes being threatened or harassed. These numbers are very concerning given that Asian Americans account for 6% of the total population in the United States, representing the fastest growing ethnic group [31]. These numbers are not negligible because disease-related anti-Asian sentiment has caused some damage—for example, an Asian American family was stabbed in March in Texas while shopping at a Sam’s Club; an Asian woman was hit on the head with an umbrella in mid-April accompanied with anti-Asian insults [5]. These types of hate crimes have caused additional fear and anxiety among Asians [7]. Our study further suggests that during a prolonged pandemic like COVID-19, we should pay careful attention to other social issues relating to the pandemic.

Second, frequent use of social media to obtain COVID-19 news was positively associated with both experience of everyday discrimination and concerns about future discrimination. This finding can be explained by a number of reasons. First, social media can be easily flooded with unconfirmed negative stereotypes, emotion-arousing information, and racist hate speech [17,32]. Therefore, those who use social media more frequently may have a higher chance of being exposed to racism-related information. Second, research has shown that social media is usually associated with a high degree of ethnic homophily, meaning that people from the same ethnic group are more likely to gather together on social media [33,34]. Our Asian participants likely had a social media network composed of a relatively high percentage of Asian friends, who may have shared much racism-related information as it had become a primary concern in the Asian community. Finally, social media algorithms present information to users based on users’ earlier behaviors [35]. It is conceivable that, through social media algorithms, those who frequently resort to social media for COVID-19 news are then exposed to more COVID-19 news, which may include reports on anti-Asian incidents (see examples in [4,5,12]). Thus, Asian participants who frequently accessed COVID-19 news on social media may have been overinformed of and hypervigilant about COVID-19–related racism, and thus reported more experiences of and concerns about discrimination. One recent study showed that Asians/Asian Americans who engaged in social media more during the pandemic reported more worry about being discriminated, likely because social media browsing during this time led to the perception that anti-Asian racism was pervasive [18], which resonates with our findings.

Third, it is noteworthy that usage of print media was also positively associated with experience of discrimination. Research has found some patterns regarding how different media might affect people’s information processing [36]. For example, it is noted that the nature of print media in dominantly delivering text-based information and allowing readers to process information at their own pace and sequence increases the chances of elaborating information in a deeper cognitive level, especially for those who have higher interest in the information [37]. On the other hand, television is more powerful in involving those who are less involved in the content because of its ability of activating multiple sensory modes [38]. Being treated as the main target of discrimination during the COVID-19 pandemic, Asians would be highly involved in racism-related news coverage. In this case, print media could facilitate more cognitive elaboration of relevant information among Asians, which might lead them to be more sensitive to the discrimination they experienced in their daily lives.

Finally, our study also indicated that those who self-identified as Chinese residing in the United States were more likely to experience discrimination and have concerns about future discrimination. It is not surprising that Chinese individuals were more sensitive to discrimination considering the close connection between the COVID-19 outbreak and China. Furthermore, we also found that younger age and a lengthier residency in the United States were both associated with experience of and concerns about discrimination. It would be worthwhile to conduct qualitative research to find out how age and the length of time spent living in a different country (and cultural context) can cause different perceptions of racial prejudice and discrimination.

Our study supports earlier reports that demonstrate the concerning state of Asian people during the pandemic. It reveals the potential psychological toll of attributing a pandemic to a particular ethnic group. Our results can be useful for public health agencies. To reduce discrimination against Asians during the pandemic, official sources and public health professionals should be cognizant of the possible impacts of amplifying stigmatizing cues in media reports on activating racial biases. Furthermore, health communication efforts can further emphasize evidence-based prevention measures of curbing the pandemic and help remedy social prejudices relating to the disease. Finally, Asian users could also be informed that using social media for COVID-19 information would associate with greater concerns about future discrimination, and thus they may consider approaching this media source with caution.

### Limitations and Future Studies

As an exploratory examination of Asians and their experienced and expected discrimination during COVID-19, the study has some limitations. First, even though this project reached Asians from 35 states in the country, the nonprobability sampling nature of this study will limit generalizations of the results to the whole Asian community. Second, our study revealed the prevalence of discrimination against Asians during COVID-19, but the nature of the study did not allow us to report whether and by how much discrimination increased since the outbreak. Scholars who have collected longitudinal data before and after COVID-19 are in a better position to present the change. Third, we used an existing scale of racial discrimination [24], which generated self-report data. Future studies can explore new ways to better assess the presence of racial discrimination, using experimental methods or attitudinal and behavioral indicators, as suggested by the National Research Council [39]. Lastly, our sample included Asians who resided in the United States during the outbreak and comprised US citizens, permanent residents, and temporary visitors. We asked our participants whether they self- identified as Chinese but did not ask the non-Chinese participants to report their original nationality. Future scholars may collect this information to analyze the potential role of nationality in influencing individual experiences during a global pandemic.

### Conclusions

Through a cross-sectional survey of Asian people in the United States, our study showed that a substantial percentage of this group has experienced and worried about some form of discrimination. Use of print and social media for COVID-19 news were both associated with a higher likelihood of experiencing discrimination, and social media use was further associated with concerns about future discrimination. Notably, our study contributes to the understanding of the media’s role in shaping racism-related perceptions of a group experiencing discrimination during a global health crisis. These findings revealed the importance of addressing discrimination in a health pandemic to protect the well-being of minority groups that are being linked to infectious diseases.

## Abbreviations

IRB: institutional review board
MTurk: Mechanical Turk
SARS: severe acute respiratory syndrome
